# The *copB* Is a Key Copper Resistance Gene in *Xanthomonas citri* pv. *mangiferaeindicae* GXBS06

**DOI:** 10.3390/genes17040408

**Published:** 2026-03-31

**Authors:** Mengmeng Tang, Meijing Qin, Yu Miao, Fengzhi Bie, Shuxian Zhong, Yongqiang He, Wei Jiang

**Affiliations:** 1 State Key Laboratory for Conservation and Utilization of Subtropical Agro-Bioresource, College of Life Science and Technology, Guangxi University, Nanning 530004, China; 18878755974@163.com (M.T.); yqhe@gxu.edu.cn (Y.H.); 2 College of Agriculture, Guangxi University, Nanning 530004, China

**Keywords:** mango bacterial angular leaf spot, *Xanthomonas citri* pv. *mangiferaeindicae*, copper resistance genes, copB

## Abstract

**Background/Objectives:** Mango bacterial angular leaf spot, caused by *Xanthomonas citri* pv. *mangiferaeindicae* (*Xcm*), is one of the most destructive bacterial diseases of mango, resulting in significant economic losses to the mango industry. Copper-based bactericides have been widely used for decades to control this disease, leading to increased copper resistance in the pathogen and heightened environmental risks. However, the copper resistance mechanisms of *Xcm* remain incompletely understood. **Methods:** In this study, we used *Xcm* GXBS06 isolated from major mango cultivars in Guangxi, China. We analyzed the homologs of known copper resistance-related genes in *Xcm* and found that these genes are relatively conserved across different strains. The functions of six important known copper resistance gene homologs in *Xcm* were investigated. Among them, five were functionally characterized by gene deletion, while the remaining one was characterized by overexpression because deletion was unsuccessful. **Results:** The result showed that *copB* is a critical copper resistance-related gene in *Xcm*. However, its deletion neither affects H_2_O_2_ tolerance nor virulence determinants such as extracellular polysaccharide production, biofilm formation, or cell motility. Additionally, it did not impact pathogenicity or bacterial growth within the host. The expression of *copB* was significantly induced at copper sulfate concentrations of 0.2 mM and 0.6 mM. **Conclusions:** These findings contribute to a better understanding of the copper resistance mechanisms in *Xcm* and provide a foundation for further studies on the biological control of this pathogen.

## 1. Introduction

Mango bacterial angular leaf spot, also known as mango bacterial canker, is a prevalent bacterial disease in mango production. It is distributed across many mango-growing regions and difficult to control, making it one of the most significant mango diseases [1]. The causal agent, *Xcm*, is a Gram-negative bacterium that primarily enters leaves through stomata and wounds, causing damage to mango leaves and fruits [2]. It can lead to severe infections in various mango cultivars, inducing characteristic angular, black lesions on leaves and water-soaked spots on fruits, ultimately resulting in defoliation and fruit drop [3], posing significant harm. During infection, *Xcm* produces substances such as cell wall-degrading enzymes, toxins, and extracellular polysaccharides that facilitate invasion of the plant [4].

Guangxi is one of China’s major mango-producing regions, and the mango industry makes a significant contribution to the local economy. In recent years, mango bacterial angular leaf spot has become increasingly frequent in most mango orchards in Guangxi, causing substantial economic losses to mango cultivation and processing [5]. For biological control of bacterial angular leaf spot disease in mangoes, we have systematically isolated prevalent strains from key cultivated mango varieties in Guangxi, China. All of the strains we isolated exhibited high copper tolerance, including *Xcm* GXBS06 [5] and *Xcm* GX07 [6], which have been previously reported, as well as *Xcm* B3 [7], another highly copper-tolerant strain isolated from Guangxi.

Since the accidental discovery in the late 19th century that the “Bordeaux mixture” (a blend of copper sulfate and lime) effectively controlled downy mildew in the vineyards of the Bordeaux region of France, copper-based compounds have been used as plant protectants for over a century. Copper ions (Cu^2+^) can non-specifically bind to various proteins and enzymes, particularly those containing sulfhydryl groups, within the cells of pathogenic microorganisms. This binding leads to their denaturation and inactivation, disrupts the integrity of cell membranes, and catalyzes the production of damaging reactive oxygen species (ROS), thereby achieving a broad-spectrum lethal effect against various pathogens, including fungi, bacteria, and oomycetes [8,9]. This “multi-targeted” mode of action [9,10] was once considered an inherent advantage that made it difficult for pathogens to develop resistance. Owing to their high efficacy, broad spectrum, long duration, and relatively low cost, copper-based fungicides/bactericides have been extensively utilized in various agricultural production systems worldwide, including orchards, vegetable farms, and cash crop plantations [10].

In living organisms, copper serves as an essential cofactor for numerous proteins; however, excess copper is toxic to organisms. Therefore, maintaining copper homeostasis is crucial for all life forms [11]. Bacteria primarily rely on efflux systems to prevent the intracellular accumulation of copper ions, thereby mitigating their toxic effects. Copper-based bactericides are the most important chemicals for controlling diseases caused by *Xanthomonas* species. Historically, to effectively manage these diseases, frequent and high-volume applications of copper-based bactericides were required. The extensive use of these bactericides has, in turn, driven the evolution of copper resistance in pathogens. For instance, copper-tolerant strains of *Xanthomonas citri* pv. *citri*, the causal agent of citrus canker, were first detected in Argentina in 1994 and subsequently found in Réunion and Martinique, France [11]. Furthermore, a survey in Florida revealed that nearly all (99.8%) tested strains of *Xanthomonas perforans*, a key pathogen responsible for bacterial spot of tomato, were tolerant to copper sulfate [12]. Consequently, investigating targets related to bacterial copper resistance and developing novel chemical agents for disease management is a promising approach.

The primary copper efflux proteins in bacteria are P1B-type ATPases. Two subfamilies of these ATPases, P1B-1 (CopA) and P1B-3 (CopB), are key proteins responsible for the export of intracellular copper ions [13]. In *Escherichia coli*, cellular copper homeostasis is primarily regulated by the Cue and Cus systems [11]. However, some copper-tolerant *E. coli* strains harbor the *pcoABCDRSE* gene cluster on plasmids. This cluster, functionally similar to the *copABCDRS* cluster in *Pseudomonas syringae*, enables adaptation to high copper concentrations. A central component of this system is PcoA. The level of copper resistance conferred by *pcoA* alone is much lower than that conferred by *pcoA* and *pcoB* together, suggesting a potential interaction between them [14,15]. In *Pseudomonas aeruginosa*, copper homeostasis is achieved through the CueR system, which regulates cytoplasmic copper levels, and the two-component CopR/S system, which controls periplasmic genes influencing copper distribution [15]. Within the CopR/S two-component system, several genes—including *pcoA*, *pcoB*, *czcCBA*, *czcR*, *czcS*, *ptrA*, *oprD*, *PA2806*, and *PA2807*—are regulated by *copRS*. Both PcoA and PcoB are outer transmembrane proteins that reduce copper levels in the periplasm, primarily through efflux and oxidation mechanisms, respectively [16].

In the genus *Xanthomonas*, the copper resistance phenotype is often attributed to plasmid-borne *cop* operons. These plasmids facilitate the horizontal transfer of resistance genes among different strains through conjugation [17]. The expression of *copA* and *copB* is primarily induced by the upstream gene *copL* in the presence of copper ions [18]. In *Xanthomonas. citri* subsp. *citri* (*Xac*) A44, *copL*, *copA*, and *copB* were identified as the most important copper resistance genes. Mutations in *copL* and *copA* reduced copper resistance to levels comparable to copper-sensitive strains, while mutation of *copB* only resulted in a partial reduction in copper resistance [19]. A study comparing *Xac* 306 under copper-treated and untreated conditions, analyzing the expression of 32 genes, revealed significant upregulation of genes related to pathogenicity and detoxification. This indicated that *copA* and *copB* are involved in the copper detoxification process in *Xac* 306 [20]. In *Xanthomonas fastidiosa*, *copA* and *copB* mutants were found to be more sensitive to copper than the wild-type strain. Gene expression of *copA* and *copB* was induced by low concentrations of copper ions (0 to 250 mM) but suppressed at high concentrations. Plant experiments showed that both genes influence copper homeostasis in the strain, which in turn affects its virulence and is modulated by environmental copper levels [17]. Current research has revealed that the copper resistance gene clusters found in different *Xanthomonas* species share high similarity with those in several other bacterial genera. Furthermore, the genetic basis for copper resistance often resides on plasmids [21,22], although chromosomal localization has also been reported in some instances [23].

In this study, using *Xcm* GXBS06 as the reference strain, we analyzed the homologous genes of known copper resistance genes in *Xcm* and functionally characterized six of these genes associated with copper resistance. The results indicated that only deletion of *copB* resulted in a highly significant reduction in copper resistance in *Xcm.*

## 2. Materials and Methods

### 2.1. Plasmids and Bacterial Cultures

The primers, plasmids, and bacterial strains used in this study are listed in Table 1. *Xanthomonas* strains were cultivated in NYG medium (liquid or solidified with 1% (*w*/*v*) agar) containing 3 g/L polypeptone (Cat# P8970, Solarbio, Beijing, China), 5 g/L beef extract (01-009, AOBOX, Hangzhou, China), 1 g/L yeast extract (Cat# LP0021B, Oxoid, Hampshire, UK), pH 7.0. The medium was supplemented with 50 μg/mL rifampicin (Rif), 25 μg/mL kanamycin (Kan), or 5 μg/mL tetracycline (Tc) as appropriate. *E. coli* strains were grown in LB medium (liquid or solidified with 1% (*w*/*v*) agar) containing 10 g/L tryptone (Cat# T8940, Solarbio, Beijing, China), 10 g/L NaCl, 5 g/L yeast extract, pH 7.0. When indicated, kanamycin (25 μg/mL) was added, and cultures were incubated at 37 °C.

### 2.2. DNA and RNA Manipulations

DNA manipulations were performed following the procedures described by Sambrook et al. [31]. Conjugation between *E. coli* and *Xanthomonas* strains was carried out as described by Turner et al. [32] Restriction endonucleases, T4 DNA ligase, and Pfu polymerase were sourced from Promega (Shanghai, China). Total RNA was extracted from *Xcm* strain cultures using the TransZol Up Plus RNA Kit (TransGen Biotech, Beijing, China) according to the manufacturer’s instructions. Reverse transcription of RNA was performed using the HiScript^®^ III RT SuperMix for qPCR (+gDNA wiper) kit (Vazyme^TM^, Nanjing, China) following the manufacturer’s protocol. Relative quantification of gene expression was conducted using the 23S rRNA gene as an internal control. The transcriptional levels of *copB* were analyzed by qRT-PCR using total RNA extracted from *Xcm* strains grown in NB medium for 24 h. SYBR green-labeled PCR fragments were amplified using primer set 3670f/r (Table 2). Three independent biological replicates were performed, and each biological replicate contained three technical replicates. The expression level of the 23S rRNA gene was used as an internal standard.

### 2.3. Construction of Deletion Mutants

In-frame deletion mutants were constructed using the homologous recombination vector pK18*mobsacB*. As an example, the ΔcopB mutant was generated as follows. A DNA fragment containing the *copB* gene with its flanking regions was amplified from *Xcm* GXBS06 genomic DNA using primer pairs 3670LF/3670LR and 3670RF/3670RR (Table 2). The two fragments were ligated into the *Eco*RI/*Xba*I and *Xba*I/*Hin*dIII sites of pK18*mobsacB*, respectively, resulting in the deletion construct, in which an internal portion of the *copB* coding region was replaced by the *Xba*I site. The construct was verified by PCR and sequencing. The recombinant plasmid was introduced into *Xcm* GXBS06 by conjugation with *E. coli* DH5α (pRK2013). Transconjugants were selected on NYG medium containing kanamycin (25 μg/mL). Single-crossover integrants were identified by PCR, and double-crossover mutants were selected on NYG medium containing 10% sucrose. Gene deletion was confirmed by PCR using primers flanking the target locus (3670F and 3670R) and by Sanger sequencing. The same strategy was applied to generate the ΔXCM1423 and ΔXCM3130-33 mutants using the respective primer pairs listed in Table 2. All deletion mutants were confirmed by PCR and sequencing.

### 2.4. Construction of Complemented Strain

The complemented strain CΔcopB was constructed using the broad-host-range vector pLAFR6. A 2268-bp DNA fragment containing the full-length *copB* gene with its native promoter region was amplified by PCR using primer pairs 3670F and 3670R (Table 2) and cloned into pLAFR6, giving a recombinant plasmid. The construct was verified by PCR and Sanger sequencing. The verified plasmid was introduced into the ΔcopB mutant by conjugation with *E. coli* DH5α (pRK2013). Transconjugants were selected on NYG medium containing tetracycline (5 μg/mL). Successful complementation was confirmed by PCR amplification of the *copB* gene using primers 3670F and 3670R, and by sequencing of the plasmid isolated from the complemented strain. The resulting strain was designated CΔcopB.

### 2.5. PCR Confirmation and Sequencing

Deletion mutants and the complemented strain were verified by PCR and Sanger sequencing. PCR was performed using primer pairs listed in Table 2. For deletion mutants, primers flanking the target locus were used; the resulting PCR products from the wild-type and mutant strains showed the expected size difference, confirming the deletion. For the complemented strain, the presence of the *copB* gene was confirmed using primers 3670F/3670R, which amplified the full-length *copB* fragment in the wild-type and complemented strains but not in the ΔcopB mutant. PCR was carried out with initial denaturation at 95 °C for 5 min; 30 cycles of 95 °C for 30 s, Tm °C for 30 s, and 72 °C for 1 min/kb; and a final extension at 72 °C for 5 min. PCR products were purified and sequenced by Sanger sequencing (Sangon Biotech, Shanghai, China) to verify the precise deletion junction (for mutants) and the correct sequence of the *copB* insert (for the complemented strain). All sequencing results were compared with the reference genome sequence.

### 2.6. Copper Resistance Assay

In this study, both qualitative plate assays and quantitative liquid culture assays were employed to evaluate the tolerance of the test strains to the heavy metal stressor CuSO_4_, thereby determining their copper resistance.

For the qualitative plate assay, test strains were cultured overnight to the mid-to-late logarithmic growth phase. The bacterial suspension was adjusted to an OD_600_ of 0.2, and 2 μL of the suspension was spotted onto NYG plates containing various concentrations of CuSO_4_. The plates were then incubated inverted at 28 °C for 3 days, after which the growth of each sample was observed.

For the quantitative liquid culture assay, test strains were cultured overnight to the mid-to-late logarithmic growth phase. The bacterial concentration was adjusted to an OD_600_ of 1.0. Then, 10 μL of this suspension was pipetted into 90 μL of liquid medium containing different concentrations of CuSO_4_ in a 96-well plate. The plate was incubated with shaking at 600 rpm for 24 h, and subsequently, measurements were taken using a microplate reader.

### 2.7. Observation of Cell Morphology by Scanning Electron Microscopy (SEM)

Test strains were cultured overnight to the mid-to-late logarithmic growth phase. Bacterial cells were harvested by centrifugation, and the pellets were washed three times with phosphate-buffered saline (PBS). The washed bacterial suspensions were then inoculated into NYG medium containing copper ions at concentrations of 0 mM, 0.5 mM, and 0.6 mM, and cultured in a shaker at 28 °C with agitation at 200 rpm until an OD_600_ of approximately 0.8 was reached. Samples were collected by centrifugation at 8000 rpm for 5 min. The supernatant was discarded, and the pellets were resuspended in PBS and washed three times by repeated centrifugation (8000 rpm, 5 min). The washed bacterial cells were fixed overnight in a 2.5% glutaraldehyde solution. After fixation, the cells were washed three times with PBS. Subsequently, small paper packets were prepared for dehydration: filter paper was cut into 4 cm × 4 cm squares. A piece of aluminum foil (approximately 2 cm^2^) was spread at one end of the filter paper square, folded evenly three times along the edge of the aluminum foil, and stapled securely to form a small packet. The concentrated bacterial suspension obtained after centrifugation was added into the small paper packet, and the open end was immediately sealed with a stapler. Gradient ethanol dehydration was then performed by sequentially immersing the paper packets containing the bacterial samples in 50%, 70%, 85%, and 95% ethanol for 15 min each, followed by two changes of 100% ethanol for 20 min each. Throughout this process, care was taken to ensure the packets were completely submerged in the ethanol solution. The packets were then dried overnight in an oven at 40 °C. Cell morphology was observed using a high-resolution field emission scanning electron microscope.

### 2.8. Identification and Evolutionary Analysis of Copper Resistance Genes in Xcm GXBS06

Based on the sequences of genes known to be associated with copper homeostasis in *Xac*, *E. coli*, and *P. aeruginosa*, a BLASTp 2.12.0 search was performed to identify their homologous genes in *Xcm* GXBS06 (GCA_045799005.1), *Xac* 306 (GCA_000007165.1), *Xanthomonas oryzae* pv. *oryzae* (*Xoo*) PXO99A (GCA_000019585.2), *Xanthomonas campestris* pv. *campestris* (*Xcc*) 8004 (GCA_045712965.1), and *Xanthomonas oryzae* pv. *oryzicola* (*Xoc*) GX01 (GCA_008370835.2). The criteria for homology were set as identity > 20%, query coverage > 50%, and an e-value < 1 × 10^−5^. The protein structural conformations were concurrently predicted using AlphaFold (https://alphafoldserver.com/) [33] and subsequently validated against a comprehensive collection of experimentally determined and AlphaFold-predicted protein structure models available through the FoldSeek server (https://search.foldseek.com/search (24 March 2026)) using FoldSeek [34] under default settings. Sequence alignment of the *copA* and *copB* genes predicted in this study was performed using MUSCLE (v 5.2) [35]. ModelTest-NG (v0.1.7) [36] was then employed to determine the best-fit models, followed by maximum likelihood phylogenetic tree inference using RAxML-NG (v1.2.2) [37] with 1000 bootstrap replicates (—seed 2 —bs-trees 1000). The models applied for *copA* and *copB* were VT+G4+F and WAG+I, respectively. All phylogenetic trees were visualized using iTOL (https://itol.embl.de) [38].

### 2.9. Statistical Analysis

All experiments were performed with three independent biological replicates. Data are presented as mean ± standard deviation (SD). Statistical analysis was performed using GraphPad Prism 9.0. Two-way analysis of variance (ANOVA) followed by Tukey’s multiple comparisons test was used to evaluate the effects of strain type and copper concentration on bacterial growth. Differences were considered statistically significant at *p* < 0.05 (*), *p* < 0.01 (**), *p* < 0.001 (***), and *p* < 0.0001 (****). For qRT-PCR analysis, relative expression levels were calculated using the 2^−ΔΔCt^ method, and comparisons between groups were performed using Student’s *t*-test.

## 3. Results

### 3.1. Xcm GXBS06 Exhibits Strong Copper Tolerance

**Figure 1:**
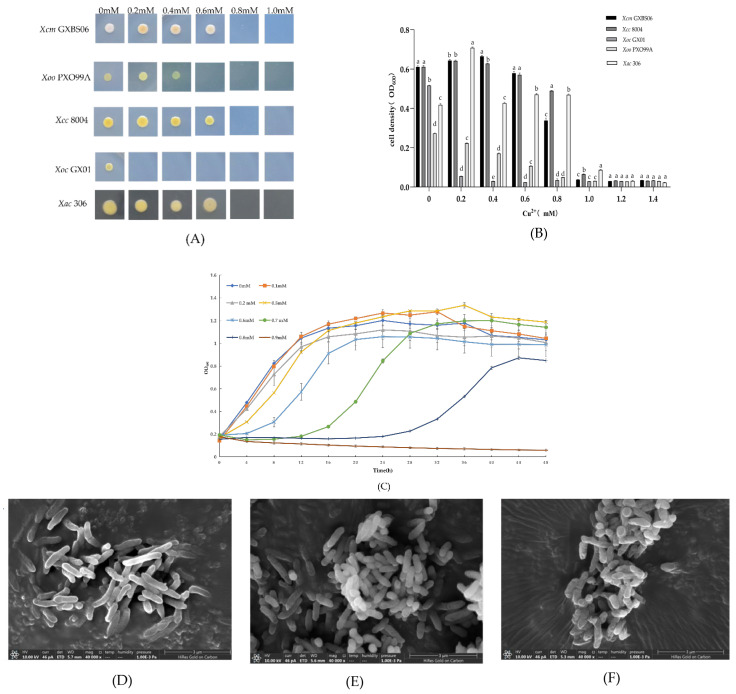
Copper resistance assay of strain *Xcm* GXBS06. (**A**) Qualitative plate assay for copper ion resistance of strains *Xcm* GXBS06, *Xcc* 8004, *Xoc* GX01, and *Xoo* PXO99A; (**B**) quantitative liquid assay for copper ion resistance of strains *Xcm* GXBS06, *Xcc* 8004, *Xoc* GX01, and *Xoo* PXO99A(different lowercase letters above the bars indicate statistically significant differences among groups at the same copper concentration (*p* < 0.05). Bars sharing the same letter are not significantly different.); (**C**) effect of different copper ion concentrations on the growth of strain *Xcm* GXBS06; (**D**) *Xcm* GXBS06 at a copper ion concentration of 0 mM (magnification, 40,000×); (**E**) *Xcm* GXBS06 at a copper ion concentration of 0.2 mM (magnification, 40,000×); (**F**) *Xcm* GXBS06 at a copper ion concentration of 0.6 mM (magnification, 40,000×).

In this study, we evaluated the copper tolerance of *Xcm* GXBS06 and several *Xanthomonas* strains preserved in our laboratory (e.g., *Xoo* PXO99A, *Xoc* GX01, *Xcc* 8004). The results showed that *Xcm* GXBS06 could tolerate up to 0.8 mM Cu^2+^ (Figure 1A,B), a tolerance level comparable to that of *Xcc* 8004 and significantly higher than that of *Xoo* and *Xoc*, indicating that *Xcm* GXBS06 possesses a notable copper-resistant phenotype.

Growth curve analysis under different copper concentrations revealed that *Xcm* GXBS06 reached its peak growth after approximately 20 h of culture in NYG medium without copper ions. Low concentrations of copper ions (0.1–0.2 mM) did not inhibit growth and even slightly promoted it. When the copper concentration was increased to 0.5–0.7 mM, early-stage growth was inhibited, but growth levels largely recovered to those of the control group in the later stage. Under 0.8 mM Cu^2+^, although growth was severely inhibited, limited proliferation could still be sustained in the later stage. At copper concentrations ≥ 0.9 mM, the strain failed to grow entirely (Figure 1C).

Scanning electron microscopy (SEM) observation revealed no significant differences in the cells of *Xcm* GXBS06 between the control group (0 mM) and the stress groups (0.2 mM, 0.6 mM) (Figure 1D–F), indicating that the cell surface structure of the strain remained relatively stable within these copper concentration ranges.

### 3.2. Evolutionary Relationship Analysis of Copper Resistance Genes in Xcm GXBS06

**Figure 2:**
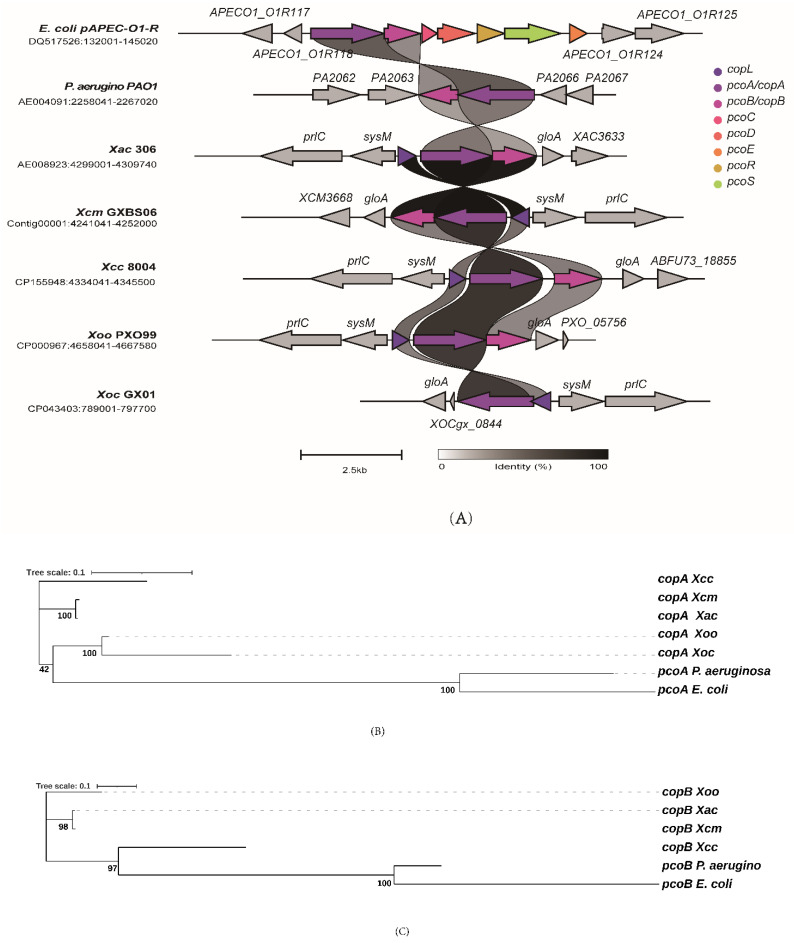
Evolutionary relationship analysis of known copper resistance genes. (**A**) Major copper resistance gene clusters in seven strains. (**B**) Phylogenetic tree of *copA* homologs. (**C**) Phylogenetic tree of *copB* homologs.

To comprehensively analyze the distribution of copper resistance-related genes in *Xcm*, sequences of genes known to be associated with copper homeostasis in *E. coli*, *P. aeruginosa*, and *Xac* were retrieved and used to identify their homologous genes in different pathogenic microorganisms (Table 3, Figure 2A).

The results indicated that the analyzed strains share a similar set of genes related to copper homeostasis. All of them contained the resistance nodulation division (RND)-type transmembrane efflux pump system *czcCBA*, the periplasmic copper chaperone *ptrA*, the multicopper oxidase *pcoA*, and the siderophore receptors *fpvA/B* (Table 3). This suggests that bacterial systems for copper homeostasis are broadly conserved, implying that these organisms may employ similar mechanisms to cope with external copper stress. Compared to *P. aeruginosa*, all the other tested strains lacked homologs of the important copper chaperone genes *copZ1/Z2*. Additionally, *E. coli* lacked *fpvA/B*. The plasmid of *E. coli* contains the *pcopABCDERS* gene cluster; *P. aeruginosa* contains *copAB* and lacks *copL*. Among several strains of the genus *Xanthomonas*, except for *Xoc*, all others contain the complete *copLAB* gene cluster, and *Xoc* was missing *copB*, which is involved in copper efflux and oxidation to reduce Cu^2+^ levels in the periplasm (Table 3, Figure 2A). Owing to the relatively low sequence identity (≥20%) between copper-related genes in the genus *Xanthomonas* and the known copper resistance genes in *E*. *coli*, the results of structural similarity comparison fail to support the presence of homologous genes for *cueR*, *pcoRS*, and *copA* in the genus *Xanthomonas*, but other homologous genes have similar structures (Table 3). Owing to the relatively low sequence identity (≥20%) between copper-related genes in the genus *Xanthomonas* and the known copper resistance genes in *E. coli*, the results of structural similarity comparison fail to support the presence of homologous genes for *cueR*, *pcoRS*, and *copA* in the genus *Xanthomonas*, but other homologous genes have similar structures (Table 3). Analysis of the flanking sequences of *copAB* revealed that, within the genus *Xanthomonas*, the downstream region of *copAB* is transcribed in the same direction as *gloA*, while its upstream region is transcribed in the opposite direction to *sysM* and *prlC*. No analogous structure exists in *E. coli* and *P. aeruginosa* as observed in the genus *Xanthomonas* (Figure 2A).

To investigate the evolutionary relationships of copper resistance genes, we constructed phylogenetic trees for *copA* and *copB*. The *copAB* phylogenetic tree (Figure 2) showed that all *copAB* sequences from *Xanthomonas* were clearly separated from the outgroup sequences from *E. coli* and *P. aeruginosa*. Among these, *copA* of *Xcm* formed a clade with that of its close relative *Xac*, with extremely short branch lengths, indicating high sequence similarity between them. This clade further formed a sister group to that of *Xcc*. Notably, *copA* from *Xoo* and *Xoc* also clustered together, but the branch length of *Xoc* was considerably longer than that of *Xoo*, suggesting that *copA* in *Xoc* might have evolved at a faster rate (Figure 2B). In contrast to *copA*, the *copB* phylogenetic tree (Figure 2C) exhibited a distinct topology. The *copB* sequences of *Xcm* and *Xac* still clustered together with extremely short branch lengths. However, this clade did not directly cluster with the *copB* of *Xcc*; instead, it formed a sister group to a larger clade comprising the outgroup sequences (*pcoB* from *E. coli* and *P. aeruginosa*) and the *copB* of *Xcc*. Additionally, the *copB* from *Xoo* was positioned at the base of the phylogenetic tree, distant from all other *Xanthomonas* and outgroup sequences, with a long branch length.

### 3.3. copB Is a Critical Copper Resistance Gene in Xcm GXBS06

**Figure 3:**
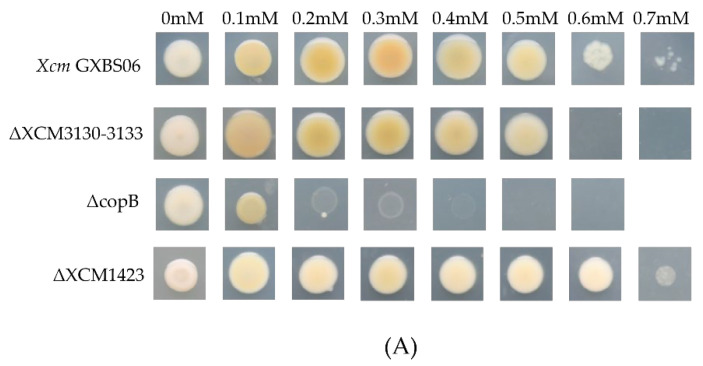

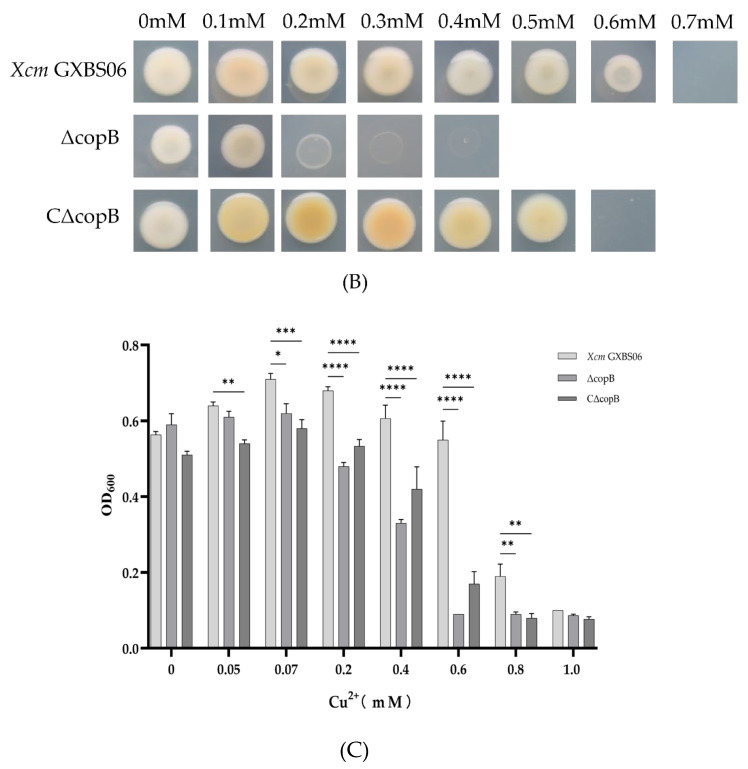
Results of copper resistance assays. (**A**) Growth of five deletion mutants on solid medium with different copper ion concentrations; (**B**) growth of *copB* on solid medium with different copper ion concentrations; (**C**) growth of *copB* in liquid medium with different copper ion concentrations. Data represent the mean ± SD of three independent biological replicates. Statistical significance was determined by two-way ANOVA with Tukey’s multiple comparisons test. * *p* < 0.05, ** *p* < 0.01, *** *p* < 0.001, **** *p* < 0.0001, compared to the wild-type strain at the corresponding copper concentration.

Among the candidate copper resistance-related genes identified through homology analysis (Table 3), six were selected for functional characterization. Deletion mutants were successfully constructed for five of these genes (*copB*, *XCM1423*, and *XCM3130-33*), while attempts to delete *XCM3671* were unsuccessful. Therefore, *XCM3671* was characterized via overexpression analysis. Subsequently, systematic resistance assays were conducted on these mutants under varying copper ion concentrations. The results showed that on solid medium, the wild-type strain could still form sparse colonies in the presence of 0.7 mM copper ions. However, among all mutants tested, ΔcopB exhibited the most significant reduction in copper resistance, failing to grow on plates containing 0.3 mM copper ions. In contrast, mutants of the other four genes showed no obvious changes in copper resistance (Figure 3A). In liquid medium supplemented with 0.2 mM copper ions, the *copB* mutant ΔcopB displayed significant growth inhibition (Figure 3B). To validate its function, a complemented strain, CΔcopB, was constructed. Copper resistance assays revealed that the copper tolerance of the ΔcopB mutant was significantly decreased compared to the wild-type strain, while the resistance level of the complemented strain CΔcopB was substantially restored to wild-type levels (Figure 3C). These results strongly suggested that *copB* is a key copper resistance gene in *Xcm* GXBS06. At copper concentrations exceeding 0.6 mM, the complemented strain did not fully recover to wild-type levels (Figure 3C).

### 3.4. copB Expression Is Significantly Upregulated Under Both Low (0.2 mM) and High (0.6 mM) Copper Ion Concentrations

To verify whether *copB* expression is induced by copper ions, the expression levels of *copB* were examined under stress conditions of 0.2 mM and 0.6 mM copper ions. qRT-PCR results showed that under low-concentration (0.2 mM) copper stress, the expression level of *copB* was significantly upregulated 90.73-fold (log_2_ fold change = 6.50) compared to the control group; under high-concentration (0.6 mM) stress, its expression level reached 40.45-fold (log_2_ fold change = 5.34) compared to that of the control group (Table 4). These results indicate that *copB* expression is strongly induced by different concentrations of copper ions.

### 3.5. Overexpression of XCM3671 Significantly Enhances Copper Tolerance in Xcm and Xoc

**Figure 4:**
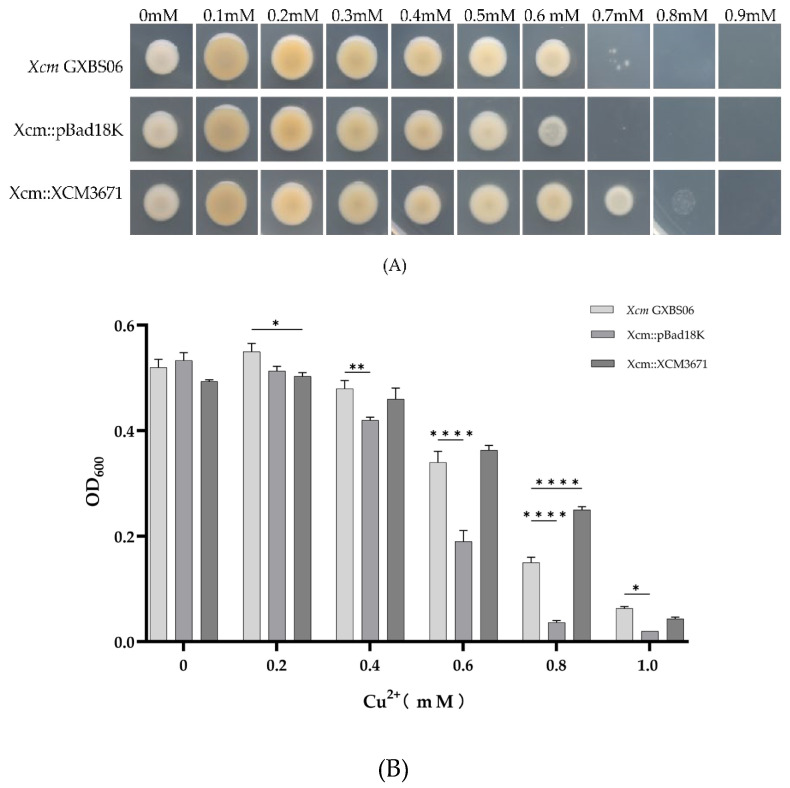

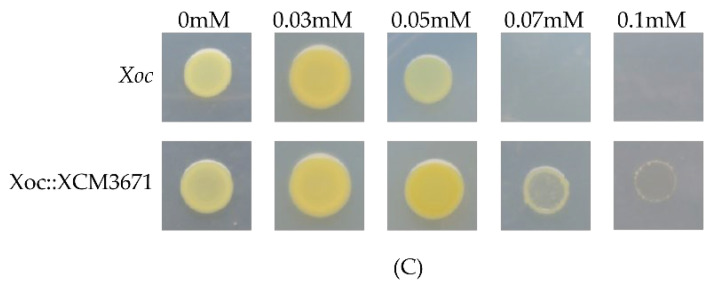
Functional characterization of *XCM3671*. (**A**) Growth of the arabinose-inducible overexpression strain of *XCM3671* on solid medium with different copper ion concentrations; (**B**) growth of the arabinose-inducible overexpression strain of *XCM3671* in liquid medium with different copper ion concentrations; (**C**) growth of the heterologous expression strain of *XCM3671* on solid medium with different copper ion concentrations. Data represent the mean ± SD of three independent biological replicates. Statistical significance was determined by two-way ANOVA with Tukey’s multiple comparisons test. * *p* < 0.05, ** *p* < 0.01, **** *p* < 0.0001, compared to the wild-type strain at the corresponding copper concentration; ns, not significant.

In *Xanthomonas* species, the function of *copB* is often associated with *copA*, which is annotated as a multicopper oxidase gene closely related to copper homeostasis. Homology analysis revealed that *copA* corresponds to locus tag *XCM3671* in *Xcm* GXBS06. Although there is currently no evidence indicating that this gene is a housekeeping gene, we attempted various mutagenesis methods, including integrative mutagenesis, insertional mutagenesis, and simultaneous deletion of both *XCM3671*(*copA*) and *copB*. However, we were consistently unable to obtain a mutant of its homologous gene, *XCM3671*. To investigate the role of *XCM3671* in copper resistance, an arabinose-inducible plasmid carrying this gene was introduced into *Xcm* and *Xoc* for overexpression. The Xcm::XCM3671 strain was able to grow on plates and in liquid medium containing 0.8 mM copper ions, exhibiting significantly enhanced copper resistance compared to the wild-type strain (Figure 4A,B). This indicates that *XCM3671* is indeed involved in copper resistance. *Xoc* possesses a homolog of *XCM3671* but lacks a homolog of *copB* (Table 3, Figure 2A), and its maximum tolerated copper concentration is only 0.05 mM, making it the most copper-sensitive strain among the *Xanthomonas* species tested to date. Compared to the *Xoc* wild-type strain, the Xoc::XCM3671 strain showed significantly enhanced copper resistance, with its maximum tolerated copper concentration increasing from 0.05 mM to 0.1 mM (Figure 4C). These results collectively demonstrate that *XCM3671* significantly enhances copper resistance in both *Xcm* and *Xoc*.

## 4. Discussion

Guangxi, China, is one of the most important mango-producing regions in the country, consistently ranking among the top three provinces for mango yield. Since none of the main cultivated mango varieties possess immunity to *Xcm*, copper-based compounds have been the primary bactericides used to limit the occurrence, development, and spread of this pathogen. Currently, all three *Xcm* (GXG07, B3, and GXBS06) strains isolated from Guangxi exhibit high copper resistance, with no copper-sensitive strains having been identified to date [5,6,7]. In-depth research on copper resistance genes is therefore crucial for the prevention and control of this disease.

Copper is an essential trace element for all life forms, participating as a cofactor in the active centers of various key enzymes, such as cytochrome c oxidase and superoxide dismutase [39,40]. However, “the dose makes the poison”, and excess copper becomes lethal. Consequently, throughout their long evolutionary history, microorganisms have necessarily developed sophisticated copper homeostasis regulatory networks to maintain a delicate balance between essentiality and toxicity.

Research indicates that the copper resistance mechanism in pathogenic bacteria is a complex, multi-gene cooperative system. It primarily includes: (1) Active Efflux Systems: Represented by the RND family efflux pump encoded by the *cusABC* gene cluster, these systems actively pump excess copper ions from the cytoplasm or periplasm out of the cell using cellular energy (e.g., proton motive force), constituting a core resistance mechanism [41]. (2) Sequestration/Storage Systems: Exemplified by the *copABCD* gene cluster, which encodes a series of proteins localized in the periplasm, inner membrane, and outer membrane [18,42,43]. For instance, CopA (a P-type ATPase) pumps excess cytoplasmic copper into the periplasm; CopC is a periplasmic copper chaperone involved in copper capture and transport; CopB is a putative outer membrane protein potentially involved in final copper efflux or sequestration [44]; and the function of CopD is not fully understood. In *P*. *syringae*, *copA* and *copB* contribute partially to copper resistance [45]. This system functions like a “temporary warehouse,” safely sequestering toxic copper ions in compartments less disruptive to cellular metabolism. (3) Oxidative Detoxification Systems: Multicopper oxidases (e.g., CueO) can oxidize the more toxic cuprous ions (Cu^+^) to the less toxic cupric ions (Cu^2+^), thereby reducing cellular damage [18]. (4) Regulatory Systems: Centered on two-component systems like CopR/S or CusR/S, these act as “radars” and “switches.” The sensor protein (CopS/CusS) perceives changes in environmental copper concentrations. Upon exceeding a threshold, it is activated and phosphorylates its cognate response regulator (CopR/CusR) [40,46]. The phosphorylated regulator then binds to promoter regions of resistance genes (e.g., *copABCD*, *cusABC*), initiating their transcription and thus building the “defensive fortifications.” Bioinformatic analysis reveals that *Xcm* lacks plasmid-borne *cop* gene clusters. Instead, it retains only an incomplete *pcop* gene cluster on its chromosome, homologous to the plasmid-borne *pcop* cluster found in *E. coli*. In most *Xanthomonas* species, copper resistance is conferred by the plasmid-borne *copLAB* gene cluster [47]. The *pcop* gene cluster does not play a primary role in *E. coli* copper resistance, where *cueR* is critical [48]. Within the genus *Xanthomonas*, the system governed by the *cop* gene cluster is a significant mechanism for maintaining copper homeostasis. Comparative analysis of *cop* operons across various *Xanthomonas* and *Pseudomonas* species revealed that the *Xanthomonas cop* operon is among the smallest, relying solely on CopA and CopB to confer substantial copper resistance, highlighting the crucial role of *copAB* in the copper resistance mechanisms of this genus [49]. Within *copLAB*, *copL* plays a minor role in copper resistance, whereas *copA* is critical [50]. In this study, we systematically mutated homologs of several important known copper resistance genes in *Xcm* GXBS06 and assessed the copper tolerance of the resulting mutants. We identified *copB* as an important copper resistance gene in *Xcm*. However, *copB* deletion did not affect extracellular amylase activity, extracellular polysaccharide production, extracellular cellulase activity, extracellular protease activity, swarming motility, swimming motility, or antioxidant capacity. It also did not impact pathogenicity or bacterial growth within the host, suggesting that its function is specifically related to copper resistance. Unfortunately, we were unable to obtain a *copA* mutant, precluding direct assessment of its role in copper tolerance. Nevertheless, overexpression of *copA* significantly enhanced copper resistance in both *Xcm* and *Xoc*, indicating that *copA* is also a copper resistance-related gene. *XCM3130/3131/3133* is predicted to constitute the *czcABC* efflux system, which could theoretically transport excess copper ions from the periplasm out of the cell. However, this system appeared non-functional for copper efflux in *Xcm* under the conditions tested, although it might play a role in the efflux of other metal ions. Additionally, this loss of function was also observed for the *cueR* homolog *XCM1423*. These findings suggest that *Xanthomonas* and *Escherichia* employ fundamentally different primary systems to cope with elevated environmental copper concentrations. According to phylogenetic analysis, the topology of *copAB* from *Xanthomonas* was clearly separated from the outgroup sequences from *E. coli* and *P. aeruginosa.* The distinct evolutionary histories of the copper resistance genes *copA* and *copB* in *Xanthomonas* are largely consistent with the species classification of the host bacteria, wherein the *X. citri* clade is separated from the *X. campestris* clade, while the *X. oryzae* clade forms an independent lineage. This pattern suggests that *copA* has primarily been transmitted vertically within the genus *Xanthomonas*, functioning as a core gene involved in basal copper homeostasis. This phylogenetic topology strongly suggests that *copB* may have undergone horizontal gene transfer (HGT). This finding is consistent with reports that copper resistance genes are often located on plasmids or mobile genetic elements, and provides a potential explanation for the substantial variability in copper resistance capacity among different species within the same genus, or even among different strains of the same species.

Due to the overall low similarity between the copper resistance-related genes of *Xanthomonas*, *E. coli* and *P. aeruginosa*, we conducted auxiliary analysis on these genes using structural similarity. Through Alphafold’s structural simulation and Foldseek’s search, *XCM1423*, *XCM2092*, *XCM2014/2013*, and *XCM2541/2542* have shown no structural similarity with CueR, CopA, and CopRS, respectively. This indicates that these genes may have different functions in *Xcm* from those in *E. coli* and *P. aeruginosa*.

For many copper-tolerant pathogenic bacteria, high concentrations of copper can lead to cell wall thickening or the formation of abnormal surface structures (such as protrusions or exudates), which represent a defensive response by the bacteria attempting to prevent copper ions from entering the cell [51]. Studies on *Xanthomonas* have shown that under copper stress, *Xac* can enter a viable but non-culturable (VBNC) state [52]. Concurrently, to counteract copper stress, bacteria may increase the synthesis of the protective layer EPS to sequester free copper ions, resulting in morphological changes such as a smoother or altered surface glossiness [53]. However, morphological changes induced by copper stress have not been observed in other studies on copper resistance within the genus *Xanthomonas*. In the present study, SEM analysis revealed no changes in the surface morphology or motility of *Xcm* under different copper ion concentrations.

In *Xac*, gene expression analysis of the *cop* operon revealed that *copAB* transcripts were detectable only when copper was added to the culture medium. The accumulation of *copAB* transcripts subsequently induced CopA and CopB, confirming the coupling of copper-induced transcription and translation [49]. In the present study, *copB* expression levels were significantly upregulated under both 0.2 mM and 0.6 mM copper ion concentrations, suggesting that *copB* expression in *Xcm* is also copper-inducible.

In some *Xanthomonas* species, *copAB* are typically located within a *cop* gene cluster. In *Xanthomonas. axonopodis* pv. *vesicatoria*, copper resistance genes are plasmid-borne, and their expression is regulated by *copL*, an open reading frame (ORF) located directly upstream of *copAB*. Notably, their expression does not depend on the two-component systems (*copRS* or *pcoRS*) [54]. In *Xanthomonas. axonopodis* pv. *manihotis*, highly conserved *copLAB* or *copABCD* gene clusters are widely distributed [55].

In the homology analysis of copper resistance genes conducted in this study, we evaluated copper tolerance in *Xcm* alongside *Xoo*, *Xoc*, *Xac*, and *Xcc*. We observed that copper tolerance among *Xanthomonas* species was generally consistent. However, as shown in Figure 1A,B, *Xoc* exhibited the weakest copper tolerance among the five strains tested, tolerating only 0.05 mM copper ions. Comparative analysis revealed that *Xoc* possesses *copA* and *copL* homologs but lacks *copB*. This observation suggests a correlation between the presence of *copB* and higher copper tolerance among *Xanthomonas* species. However, whether the absence of *copB* is the direct cause of the low copper tolerance in *Xoc* requires further experimental validation, as other genetic differences among these strains may also contribute to the observed phenotype. Nonetheless, these findings support that *copB* plays an important role in copper resistance within the genus *Xanthomonas*. Furthermore, the function of *copA* in *Xoc* may be comparatively weaker than that of its homolog in *Xac*, which could also explain why overexpression of *copA* in *Xcm* only increased its copper tolerance to 0.1 mM. This highlights that substantial functional differences exist among homologous genes across different bacterial strains.

## 5. Conclusions

In this study, using the copper-tolerant strain *Xcm* GXBS06, we identified five potential copper resistance-related (plus one analyzed by overexpression) genes through homology analysis. Ultimately, the crucial copper resistance gene *copB* was confirmed. *copB* encodes the copper efflux protein CopB, and mutation of *copB* led to a significant decrease in the copper resistance of this strain, demonstrating that *copB* is an important copper resistance gene in *Xcm* GXBS06. These findings provide a foundation for further studies on copper resistance mechanisms and the management of *Xcm*.

## Figures and Tables

**Table 1:** Bacterial strains and plasmids used in this study.

Strains or Plasmids	Relevant Characteristics	Reference or Source
*X* *cm*		
*Xcm* GXBS06	*Xcm* wild-type strain, Rif ^r^	[5]
ΔcopB	As *Xcm* GXBS06, but *copB* (*XCM3670)* gene deleted, non-polar effect, Rif ^r^	This study
CΔcopB	ΔcopB harboring a recombinant plasmid derived from the full length of *copB* cloned into the promoterless plasmid pLAFR6., Rif ^r^, Tc ^r^	This study
ΔXCM3130-33	As *Xcm* GXBS06, but *XCM3130-33* gene deleted, non-polar effect, Rif ^r^	This study
ΔXCM1423	As *Xcm* GXBS06, but *XCM1423* gene deleted, non-polar effect, Rif ^r^	This study
*Xcm*::XCM3671	*Xcm* GXBS06 harboring a recombinant plasmid derived from the full length of *XCM3671* cloned into the promoterless plasmid pLAFR6., Rif ^r^, Tc ^r^	This study
*Xcm*::pBad18K	*Xcm* wild-type strain transformed with the empty vector pBad18K, Rif ^r^, Kan ^r^	This study
*Xanthomonas oryzae pv. oryzicola (Xoc)* GX01	*Xoc* GX01 wild-type strain, Rif ^r^	[24]
*Xoc*::XCM3671	*Xoc* GX01 harboring a recombinant plasmid derived from the full length of *XCM3671* cloned into the promoterless plasmid pLAFR6., Rif ^r^, Tc ^r^	This study
*Xanthomonas oryzae pv. oryzae (Xoo)* PXO99A	*Xoo* PXO99A wild-type strain, Rif ^r^	[25]
*Xanthomonas campestris* pv. *campestris* (*Xcc)* 8004	*Xcc* 8004 wild-type strain, Rif ^r^	[26]
*E*. *coli*		
DH5α	Φ80△*lacZM15 recA1 endA1 deoR*, Kan ^r^	[27]
2013	Helper strain, Kan ^r^	This study
plasmids		
pK18*mobsacB*	pUC18 derivative, *lacZ*, *sacB*, Kan ^r,^ *mob* site. Allelic exchange vector (Suicidal vector carrying *sacB* gene for mutagenesis) ^r^	[28]
pLARFJ6	A promoterless derivative of pLAFR3, Shuttle plasmid, Tc ^r^	[29]
pBad18K	L-arabinose-inducible expression plasmid, Kan ^r^	[30]

^r^: resistant

**Table 2:** PCR Primers Used in This Study.

Primer	Sequence ^a^ (5′-3′)	Direction and Use ^b^
3130-33LF	GAATTCTGCTGCGCGCCAGACCGTGC	F, mutant construction and confirmation
3130-33LR	TCTAGAGAGGGTGTCTCCGGAATCGG	R, mutant construction
3130-33RF	TCTAGAGCGGATGCACGGCGTGGTTG	F, mutant construction
3130-33RR	AAGCTTCGCGGAAACACGGAGCTTCA	R, mutant construction and confirmation
3130-33F	GTTCCGCGACTGGGATGTGGTGTT	F, mutant confirmation
3130-33R	TCGGATTCCAGCGGCGAATA	R, mutant confirmation
3670LF	GAATTCCTGATCGACATGCGCAGCAAT	F, mutant construction and confirmation
3670LR	TCTAGAGCGAAAGCGGCTCATGCTTC	R, mutant construction
3670RF	TCTAGAGGTACCGCGGTTGGCCCTCTCC	F, mutant construction
3670RR	AAGCTTCGAAACGTGCGCAGGCGGCA	R, mutant construction and confirmation
3670F	GAATTCATGAGCCGCTTTCGCATGCA	F, mutant confirmation and complemented strain construction
3670R	TCTAGATCAAAACCAAACGCGCACTC	R, mutant confirmation and complemented strain construction
3671LF	GAATTCTTCGTGTTGGAAGCCTCC	F, mutant construction and confirmation
3671LR	TCTAGATGGAAGCATGAGCCGCTTT	R, mutant construction
3671RF	TCTAGAATCGAAAGACATGACATCT	F, mutant construction
3671RR	AAGCTTCCTGCTGCTGTGCCTGTGCCT	R, mutant construction and confirmation
3671F	GAATTCATGTCTTTCGATCCCCCGTT	F, mutant confirmation and overexpression Strain construction
3671R	AAGCTTTCATGCTTCCACCCGCACTT	R, mutant confirmation and overexpression Strain construction
3670f	AGCCAAGTTCGACCCGTT	F, CopB RT-qPCR primer
3670r	ACTGGCGCCCTACAAGTT	R, CopB RT-qPCR primer

^a^ Added restriction enzyme sites are underlined. ^b^ F, forward direction; R, reverse direction.

**Table 3:** Comparative analysis of the copper resistance genes in phytopathogenic bacteria.

Strains	TF ^a^	CYTO-C ^a^	2CS ^a^	P-Type ^a^	RND ^b^	Peri-C ^b^	MCO ^b^	Sidero ^b^	Other ^b^
*P. aeruginosa*	*cueR*	*copZ1* *copZ2*	*copRS*	*copA1,* *copA2*	*czcCBA*	*ptrA* *azu*	*pcoA*	*fpvA* *fpvB*	*pcoB*
*E. coli*	*cueR*	N/A	*pcoRS* *cusRS*	*copA*	*cusCFBA*	*pcoE* *pcoC* *cusF*	*pcoA* *cueO*	*N/A*	*pcoB* *pcoD*
*Xac*	*XAC3000*	N/A	*XAC0325/0326* *XAC0834/0835*	*XAC0757*	*XAC4162* *XAC4161* *XAC4160*	*XAC4322*	*XAC3630*	*XAC3498*	*XAC3631* *copL*
*Xcm*	*XCM1423*	N/A	*XCM2542/2541 XCM2014/2013*	*XCM2092*	*XCM3130* *XCM3131* *XCM3133*	*XCM3906*	*XCM3671*	*XCM3812*	*XCM3670* *XCM3672*
*Xoo*	*PXO_02196*	N/A	*PXO_04836/04835* *PXO_02836/02837*	*PXO_04386*	*PXO_00707* *PXO_00705* *PXO_00706*	*PXO_02282*	*PXO_03132*	*PXO_01148*	*PXO_03131* *PXO_03133*
*Xoc*	*XOCgx_3530*	N/A	*XOCgx_3291/3292* *XOCgx_4037/4036*	*XOC4118*	*XOCgx_2403* *XOCgx_2401* *XOCgx_2402*	*XOCgx_1068*	*XOCgx_0845*	*XOCgx_4723*	*N/A* *XOCgx_0846*
*Xc*	*ABFU73_06540*	N/A	*ABFU73_20535/20540* *ABFU73_17805/17800*	*ABFU73_18215*	*ABFU73_21305* *ABFU73_21300* *ABFU73_21295*	*ABFU73_04520*	*ABFU73_18840*	*ABFU73_03305*	*ABFU73_18845* *ABFU73_18835*

Comparative analysis of the organization of copper resistance genes in *P. aerugino* PAO1 (GCA_000006765.1), *E. coli* APEC 01 (GCA_000014845.1), *Xac* 306(GCA_000007165.1), Xcm GXBS06(GCA_045799005.1), *Xoo* PXO99A(GCA_000019585.2), and *Xoc* GX01 (GCA_008370835.2). TF: transcription factor; CYTO-C: cytoplasmic copper chaperone; 2CS: copper sensing two-component systems; P-type: P-type copper ATPase; RND: resistance nodulation division-type transmembrane efflux pump; Peri C: periplasmic copper chaperone; MCO: multicopper oxidase; Sidero: siderophores; N/A: not applicable. ^a^, while the genes exhibit similarity at the sequence level, their protein structures show low similarity. ^b^, both comparison of sequence and protein structure all support that they are homologous genes.

**Table 4:** Gene expression levels of *copB* under different copper ion concentrations.

Gene Name	Predicted Product	Fold Change (0.2 mM/0 mM)	log_2_ Fold Change (0.2 mM/0 mM)	Fold Change (0.6 mM/0 mM)	log_2_ Fold Change (0.6 mM/0 mM)
*copB*	Copper resistance protein B precursor (*copB*)	90.73	6.50	40.45	5.34

## Data Availability

The original contributions presented in this study are included in the article. Further inquiries can be directed to the corresponding author.
